# SG-APSIC1049: Immunogenicity of Gam-COVID-Vac and Sinopharm BBIBP-CorV vaccines in seropositive and seronegative adults

**DOI:** 10.1017/ash.2023.15

**Published:** 2023-03-16

**Authors:** Igor Stoma, Katsiaryna Korsak, Evgenii Voropaev, Olga Osipkina, Aleksey Kovalev

**Affiliations:** Gomel State Medical University, Gomel, Belarus; Gomel State Medical University, Belarus; Gomel state Medical University, Gomel, Belarus; Gomel, Gomel State Medical University, Gomel, Belarus; Gomel State Medical University, Gomel, Belarus

## Abstract

**Objectives:** Data comparing the immunogenicity of Sputnik-V and Sinopharm vaccines in seropositive and seronegative groups are lacking. We compared the immunogenicity of Sputnik-V (Gam-COVID-Vac) and Sinopharm (BBIBP-CorV) vaccines in seronegative and seropositive groups. **Methods:** In total, 60 adults participated the study. The immune response after vaccination was assessed using enzyme immunoassay. IgG levels were measured in all participants at 3 time points: before vaccination, 42 days after the first vaccine dose, and 6 months after the first vaccine dose. The results of the SARS-CoV-2 antibody test were quantified according to the WHO First International Standard and expressed in international units (BAU per mL). **Results:** The study participants were divided into 2 groups: 30 people (50%) were vaccinated with Sputnik-V (Gam-COVID-Vac) and 30 people (50%) were vaccinated with Sinopharm (BBIBP-CorV). The groups had no difference in sex composition. The highest antibody levels were observed 42 days after vaccination in both the seronegative group (*P* = .006) and the seropositive group (*P* < .001). At 6 months after vaccination, the IgG value declined much farther among the seronegative group (*P* = .003) compared to those who had recovered from COVID-19 before vaccination. However, the “hybrid immunity” generated by the Sputnik-V vaccine had greater strength and duration (*P* < .001). **Conclusions:** This study showed that IgG levels in vaccinated individuals who previously recovered from SARS-CoV-2 infection (“hybrid immunity”) were higher than in SARS-CoV-2–naïve individuals. In a comparative part of the study, the Sputnik-V vaccine had greater strength and duration of immune response across the 6-month observation period (*P* < .001).

